# Cerebello‐Cortical Control of Tremor Rhythm and Amplitude in Parkinson's Disease

**DOI:** 10.1002/mds.28603

**Published:** 2021-04-01

**Authors:** Rick C. Helmich, Kevin R.E. Van den Berg, Pattamon Panyakaew, Hyun J. Cho, Thomas Osterholt, Patrick McGurrin, Ejaz A. Shamim, Traian Popa, Dietrich Haubenberger, Mark Hallett

**Affiliations:** ^1^ Department of Neurology Radboud University Medical Centre, Donders Institute for Brain, Cognition and Behaviour Nijmegen The Netherlands; ^2^ Human Motor Control Section, National Institute of Neurological Disorders and Stroke, National Institutes of Health Bethesda Maryland USA; ^3^ Chulalongkorn Centre of Excellence for Parkinson's Disease & Related Disorders, Department of Medicine, Faculty of Medicine Chulalongkorn University and King Chulalongkorn Memorial Hospital, Thai Red Cross Society Bangkok Thailand; ^4^ Kaiser Permanente Mid‐Atlantic States Largo Maryland USA; ^5^ MidAtlantic Permanente Research Institute Rockville Maryland USA; ^6^ Defitech Chair of Clinical Neuroengineering, Center for Neuroprosthetics and Brain Mind Institute Swiss Federal Institute of Technology (Valais), Romand Rehabilitation Clinic Sion Switzerland; ^7^ Defitech Chair of Clinical Neuroengineering, Center for Neuroprosthetics and Brain Mind Institute Swiss Federal Institute of Technology Geneva Switzerland; ^8^ Clinical Trials Unit, Office of the Clinical Director, National Institute of Neurological Disorders and Stroke, National Institutes of Health Bethesda Maryland USA

The pathophysiology of Parkinson's disease (PD) tremor involves both the basal ganglia and a cerebello‐thalamo‐cortical circuit.^1^, ^2^ It remains unclear how tremor rhythm and amplitude emerge from these circuits and whether these mechanisms depend on tremor phenotype. Previous data suggest that the cerebellum is specifically involved in PD postural tremor.^3^ However, different postural tremor types (re‐emergent or pure postural tremor)^4^ were included, and region‐specific effects on tremor amplitude were not assessed. Here, we investigated the role of the motor cortex (M1) and cerebellum in generating rhythm versus amplitude of PD rest tremor compared with re‐emergent tremor.

We tested the effect of single‐pulse transcranial magnetic stimulation (TMS) on tremor rhythm (tremor reset index [TRI]) and tremor power (electromyography) in 14 patients with PD (Table S1; Appendix S1). All patients had rest tremor and electrophysiologically proven re‐emergent tremor: wrist extension suppressed tremor amplitude for up to 3000 milliseconds (*F*_1,20_ = 11.7, *P* < 0.001; part.η^2^ = 0.47; Fig. 1A,B). Cerebellum‐TMS reset re‐emergent tremor, but not rest tremor (*t*_13_ = 2.1, *P* = 0.026; Cohen's *d* = 0.57; TRI vs. 0 [re‐emergent tremor: *t*_13_ = 3.0, *P* = 0.010; rest tremor: *t*_13_ = 1.0, *P* = 0.33]; Fig. 1C–E). In re‐emergent tremor, the TRI after cerebellum‐TMS decreased with subsequent tremor bursts (1–5 after TMS), indicating transient resetting (time: *F*_4,52_ = 3.61, *P* = 0.011; part.η^2^ = 0.22; Table S2). M1‐TMS, but not cerebellum‐TMS, reduced tremor power for both rest tremor and re‐emergent tremor (site × time interaction: *F*_8,104_ = 8.77, *P* < 0.001; part.η^2^ = 0.40; Fig. 1F,G [no 3‐way interaction with tremor type]). Specifically, M1‐TMS reduced tremor power up to 1500 milliseconds in both tremor types (rest tremor, time: *F*_8,104_ = 8.17, *P* < 0.001; part.η^2^ = 0.39; re‐emergent tremor, time: *F*_8,104_ = 13.24, *P* < 0.001, part.η^2^ = 0.50), whereas cerebellum‐TMS did not influence tremor power (*F* < 1.4).

**FIG. 1:**
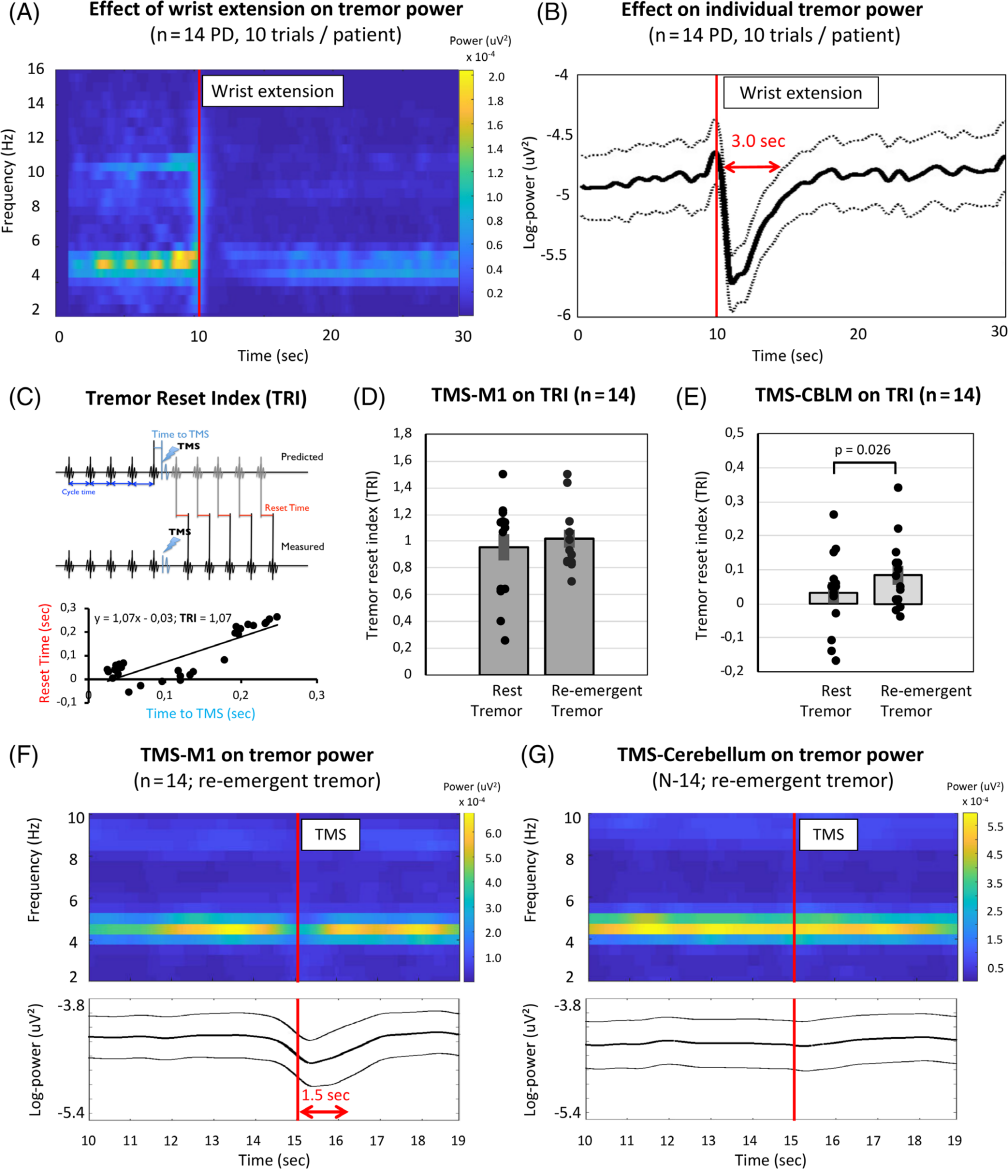
Effects of TMS on rhythm and power of rest versus re‐emergent tremor. (**A**) Average TFR of EMG power. The red line indicates voluntary wrist extension. This transiently reduces tremor power at ±5 Hz. (**B**) Average log‐transformed tremor power (from EMG) at individual re‐emergent tremor frequency (± SEM). The red arrow indicates a significant drop in tremor power (up to 3.0 seconds after TMS). (**C**) The TRI is the slope of the regression line between “time to TMS” and “reset time” (over multiple trials in each individual), here shown for 1 patient (M1‐TMS, re‐emergent tremor). (**D**,**E**) TRI (mean ± SEM) for M1 stimulation (**D**) and cerebellum stimulation (**E**) during rest and posturing. (**F**,**G**) Effect of TMS over M1 (**F**) and the cerebellum (**G**) on re‐emergent tremor. Effects for rest tremor are similar (supplement). Upper panels show the average TFR of EMG tremor power (n = 14); lower panels show the average (± SEM) log‐transformed tremor power (derived from EMG) over time at individual tremor frequencies (n = 14). Red arrow indicates a significant drop in tremor power for up to 1.5 seconds after TMS. APB, abductor pollicis brevis; CBLM, cerebellum; ECR, extensor carpi radialis; EMG, electromyography (from muscle showing clearest tremor in each patient); FCR, flexor carpi radialis; FDI, first dorsal interosseus; M1, primary motor cortex; PD, Parkinson's disease; SEM, standard error of mean; TFR, time‐frequency representation; TMS, transcranial magnetic stimulation; TRI, tremor reset index. [Color figure can be viewed at wileyonlinelibrary.com]

Our findings suggest that the cerebellum is part of the oscillator controlling the rhythm of re‐emergent tremor, but not rest tremor. Compared with previous data, the TRI after cerebellum‐TMS was smaller (0.1 vs. 0.5), and tremor reset was transient instead of permanent.^3^ This may relate to the postural tremor types included (here, re‐emergent tremor; previously, all postural tremors),^3^ to the stimulation intensity (here, 56% stimulator output; previously, 68%),^3^ or both. Re‐emergent tremor and resting tremor have been hypothesized to be a continuum (“tremor of stability”),^2^ and they share clinical features.^5^ However, re‐emergent tremor has as smaller dopamine response and slightly higher frequency than rest tremor.^4^ Our data suggest that these differences may be explained by the cerebellum, which comes in with voluntary movement and transiently modulates the tremor oscillator and possibly tremor frequency, while the fundamental character of the tremor remains unchanged.

Our data further suggest that M1, but not the cerebellum, controls tremor amplitude, independent of tremor phenotype. This finding is in line with previous data.^6^ M1‐TMS effects on tremor power were shorter compared to wrist extension (1500 vs. 3000 milliseconds), suggesting that mechanisms involved in voluntary actions may have an additional role in tremor suppression. TMS pulses were given at intensities that produce motor‐evoked potentials, so the effects may be driven in part by somatosensory afferents related to small muscle twitches. Intriguingly, thalamus interventions effectively reduce PD tremor amplitude,^7^ while cerebellum‐TMS did not. This may suggest that the effects of thalamus interventions are not (only) explained by the interruption of cerebello‐thalamo‐cortical projections, but potentially also by the interruption of cortico‐thalamo‐cortical projections.^8^

## Full financial disclosures for the previous 12 months

R.C.H. is supported by the Netherlands Organization for Scientific Research (VENI Grant 9167077) and by The Michael J. Fox Foundation (Grant 15581). He received honoraria (for scientific lectures) from the International Parkinson and Movement Disorder Society. He is handling editor at Neuroimage: Clinical and serves on the Clinical Advisory Board of Cadent Therapeutics. K.R.E.v.d.B., P.P., H.J.C., T.O., P.M., and T.P. have nothing to disclose. E.A.S. is principal investigator on multiple studies, including the PROSPECT study funded by Cala Health Inc, the ADVANCE study funded by Allergan, Inc, and GP2 funded by The Michael J. Fox Foundation. All funding was awarded to The Mid‐Atlantic Permanente Research Institute. He is also part of the National Institute of Neurological Disorders and Stroke Intramural Research Program as a special volunteer. D.H. is a full‐time employee of Neurocrine Biosciences. The work outlined in this article has been performed in the capacity of Dr. Haubenberger's prior employment at the National Institute of Neurological Disorders and Stroke Intramural Research Program and is unrelated to his current employment. M.H. is an inventor of patents held by the National Institutes of Health (NIH) for an immunotoxin for the treatment of focal movement disorders and the H‐coil for magnetic stimulation; in relation to the latter, he has received license fee payments from the NIH (from Brainsway). He is on the medical advisory boards of CALA Health and Brainsway (both unpaid positions). He is on the editorial boards of approximately 15 journals and receives royalties and/or honoraria from publishing from Cambridge University Press, Oxford University Press, Springer, and Elsevier. He has research grants from Medtronic, Inc. for a study of deep brain stimulation for dystonia and CALA Health for studies of a device to suppress tremor.

## Author Roles

(1) Research Project: A. Conception, B. Organization, C. Execution; (2) Statistical Analysis: A. Design, B. Execution, C. Review and Critique; (3) Manuscript: A. Writing of the First Draft, B. Review and Critique.

R.C.H.: 1A, 1B, 1C, 2A, 2B, 3A

K.R.E.v.d.B.: 1B, 1C, 2B, 3A

P.P.: 1A, 1B, 1C, 3B

H.J.C.: 1A, 1B, 1C, 3B

T.O.: 1A, 1B, 1C, 3B

P.M.: 1A, 1B, 1C, 3B

E.A.S.: 1A, 1B, 3B

T.P.: 1A, 1B, 1C, 3B

D.H.: 1A, 1B, 3B

M.H.: 1A, 1B, 2C, 3B

## Supporting information

**AppendixS1**. Supporting Information.
