# Supplementary material for: Cerebello‐Cortical Control of Tremor Rhythm and Amplitude in Parkinson's Disease
Source: Mov Disord. 2021 Apr 1;36(7):1727–9. doi: 10.1002/mds.28603 (PMC8359958; doi:10.1002/mds.28603)
Supplement: Supplementary file 1 — AppendixS1. Supporting Information. [file MDS-36-1727-s001.pdf]

Cerebello-cortical control of tremor rhythm and amplitude in Parkinson's disease

Rick C. Helmich, *MD, PhD*[1,2], Kevin R.E. van den Berg, *BSc*[1,2], Pattamon Panyakaew, *MD*[2,3], Hyun Joo Cho, *MD*[2], Thomas Osterholt, *BSc*[2], Patrick McGurrin, *PhD*[2], Ejaz Shamim, *MD*[4], Traian Popa, *MD, PhD*[5,6], Dietrich Haubenberger, *MD*[7], and Mark Hallett, *MD, DM*[2]

TABLE OF CONTENTS

SUPPLEMENTARY METHODS 2
SUPPLEMENTARY RESULTS 6
SUPPLEMENTARY DISCUSSION 7
SUPPLEMENTARY FIGURES 11
SUPPLEMENTARY TABLES 13
REFERENCES 15

We tested three hypotheses: (i) the cerebellum resets re-emergent postural tremor, but not resting tremor[1]; (ii) the cerebellar involvement in re-emergent tremor is time-locked to the onset of the tremor, and increases with a larger delay to tremor onset (when somatosensory afferents stabilize the tremor rhythm)[2,3]; (iii) TMS over M1, but not the cerebellum, influences tremor power across both PD tremor phenotypes[4].

SUPPLEMENTARY METHODS

Study population

We included 14 patients diagnosed with idiopathic PD according to UK Brain Bank criteria, with a marked tremor of at least one arm, defined as ≥2 points on both the resting *and* postural tremor item of the Movement Disorders Society Unified Parkinson's Disease Rating Scale (MDS-UPDRS) part III. Clinical characteristics are shown in Table S-1. Patients were measured OFF-medication, *i.e.* >12 hours after the last dose of immediate release levodopa, and were asked to withdraw from all other medications that would affect tremor (e.g. beta blockers) at least 5 half-lives before participating in this study. Exclusion criteria included problematic alcohol use (defined as ≥8 on the WHO AUDIT) and psychiatric or neurological comorbidity. Patients with ferromagnetic metal in the cranial cavity (or eye) or implanted electronic devices were excluded. This study was approved by the institutional review board of the National Institutes of Health and all subjects provided written informed consent before participation in this study.

Tremor recordings

All patients were clinically assessed using the MDS-UPDRS part III and the Tremor Research Group Essential Tremor Rating Assessment Scale (TETRAS). We measured arm tremor using surface EMG of the *First Dorsal Interosseus* (FDI), *Abductor pollicis brevis* (APB), *Extensor carpi radialis* (ECR), and *Flexor carpi radialis* (FCR) muscles of the (clinically) most-affected side. For subsequent analyses, we selected the muscle with the clearest tremor bursts. A tri-axial accelerometer (Kistler, Eemnes, the Netherlands) was placed on the dorsal surface of the same hand, with the z-axis perpendicular to the dorsal surface of the hand. EMG and accelerometry data was processed using Fieldtrip[5]. Preprocessing of EMG included band-

pass filtering (20-250 Hz), rectification, and band-pass filtering (2-16 Hz)[6]. Accelerometry data were band-pass filtered (2-16 Hz). These data were then used to calculate: (1) tremor frequency; (2) fluctuations in tremor power, time-locked to voluntary movement or TMS pulse; (3) TRI. To calculate the tremor frequency, we computed time frequency representations (TFR) between 2 and 16 Hz in steps of 0.1 seconds using a Hanning taper with a duration (time interval) of 5 seconds, resulting in a 0.2 Hz spectral resolution. To quantify tremor power, we performed a separate TFR analysis using a Hanning taper of 2 seconds, resulting in a 0.5 Hz spectral resolution, and extracted tremor power at each patient's individual peak tremor frequency. We applied a log-transformation to tremor power values, given the log-linear relationship between tremor power and clinical rating scales[7]. The procedure to calculate the TRI is outlined below.

Postural tremor phenotype

For each individual patient, we verified that there was re-emergent postural tremor, *i.e.* a tremor frequency similar to that of resting tremor (delta $\leq$ 1Hz) and a reduction in tremor power during transition from rest to postural tremor[6]. More specifically, we recorded resting tremor for 10 seconds, after which patients extended their wrist in response to a tone and maintained that posture for 60 seconds. This was done 10 times for each patient, and the average tremor power time course was calculated. We also measured tremor at rest, during posturing (outstretched, horizontal position, supported at the elbows), and during slow movements (wrist flexion/extension), for three trials of 60 seconds each. The frequency of each patient's resting and postural tremor is shown in Table S-1.

Transcranial magnetic stimulation

Two Magstim 200^2 units (Magstim, Dyfed, United Kingdom) were used to stimulate M1 and the cerebellum. M1 contralateral to the recorded side was stimulated with a regular 70 mm figure-of-eight–shaped coil, with the orientation of the coil tangential to the scalp with the handle pointing backwards and laterally at a 45-degree angle away from midline. We chose the location that evoked the largest motor evoked potentials (MEPs) in the contralateral FCR muscle. Input-output curve (IOC) parameters were obtained by delivering single TMS pulses at intensities in random order from 0 to 100% at 5% intervals of stimulator output at an inter-pulse interval of 10 seconds. Two pulses were delivered at each intensity. MEP

amplitudes were plotted against the corresponding stimulation intensity, and the value of the estimated resting motor threshold (RMT) and suprathreshold (S50) were obtained from the IOC, as done before [8,9]. The S50 was used to standardize our TMS stimulation intensity between individuals. The S50 is the intensity which is halfway between 0 and what is possible at that site of stimulation (in terms of MEP amplitude). The stimulus intensity used for resetting tremor at M1 was S50.

For cerebellar stimulation, a double-cone coil was used, with the coil positioned 3 cm lateral to the inion on the line joining the inion and external auditory meatus on the side ipsilateral to the recorded muscle. We stimulated with an intensity of 5% of stimulator output below the active motor threshold (AMT) of the pyramidal tract (which was tested with the coil centered at the inion). Subjects rapidly abducted the index finger to obtain phasic contractions of the FDI. AMT was defined as the minimum intensity required to elicit MEPs of >50uV above the background in 50% of stimulation. The coil current direction was downward to induce upward current in the cerebellar cortex. A Brainsight neuronavigation system (Rogue Research, Montreal, Canada) was used to maintain the stimulation target constant during stimulation.

Experimental design

We investigated the effect of TMS applied over M1 versus cerebellum on resting versus postural tremor in each patient (n=40 trials for each condition; pseudo-randomized in order, i.e. counter-balanced between patients, such that we alternatingly started with M1 or cerebellum stimulation). For resting tremor, we stimulated with a fixed interval of 10 seconds between TMS pulses. For postural tremor, TMS pulses were delivered time-locked to the onset of the tremor. That is, a first TMS pulse (early condition) was delivered 2s after tremor onset (which was identified using online visual and electrophysiological monitoring by viewing the accelerometry signal and manually delivering the TMS pulse), and a second TMS pulse (late condition) was delivered 10 seconds after the first TMS pulse. Each postural tremor trial lasted 20 seconds (5 seconds pre-TMS and 5 seconds post-TMS); we aimed to collect 20 trials for both the early and the late condition. For each of the four experimental conditions (M1-rest, cerebellum-rest, M1-posture, cerebellum-posture), we calculated the effect of TMS on tremor rhythm (TRI; $\leq$5 tremor bursts post-TMS) and tremor power (quantified with log-transformed EMG power at tremor frequency, $\leq$4 seconds post-TMS).

Tremor Reset Index

We used the same procedures as described before[1,10,11]. First, the TMS artifact and the MEP (for M1 stimulation) were removed from the EMG data. Then, the parameters of tremor reset were calculated from the timing of the tremor bursts (using a peak-selection routine in Matlab, with visual confirmation). Time to TMS is the interval from the peak time of the last tremor burst before stimulation to the TMS artifact. We calculated the average cycle length by averaging the interval between the peak amplitude of each EMG burst of the first five tremor bursts immediately before TMS. This cycle length was then used to calculate the expected time for the five subsequent tremor bursts that would have occurred without TMS. The reset time was defined as the interval between the actual (measured) and expected tremor burst. The TRI was calculated as the average slope of a regression line between reset time and time to TMS. This was done separately for each of the five tremor bursts after TMS. That is, we calculated each of the parameters necessary to obtain the TRI separately for each of the five tremor bursts. The regression line was based on all available trials for each condition, which differed between subjects based on the quality of the recording, tremor amplitude, and presence/absence of tremor during a trial. We only included subjects when at least 5 trials were available per condition (Table S-2). Tremor reset is absent if the TRI value is 0 and complete if the TRI value is 1. We also tested whether the TRI differed between the five subsequent tremor bursts after TMS. A similar TRI over subsequent bursts (permanent resetting) suggests that oscillatory activity is set to a new fixed point in its cycle, showing that the oscillator itself is affected[10,12]. Alternatively, TRI decreasing over subsequent tremor bursts (transient resetting) suggests that the oscillator is modulated but not altered.

Statistical analyses

First, we compared tremor frequency at rest versus posture using paired-sample t-tests. Second, we tested the effect of voluntary wrist extension on tremor power. Thus, we compared tremor power at baseline (averaged between 3 to 1 seconds before wrist extension) with tremor power values after wrist extension (in 500-ms-steps up to 10 seconds after wrist extension), using a repeated measures ANOVA with factor TIME (baseline vs. 20 post-movement 500-ms-bins). Third, we tested the effect of stimulation SITE (M1 versus

cerebellum) and TREMOR TYPE (rest, postural) on the TRI, using a 2x2 repeated measures ANOVA. Given that we had the *a priori* hypothesis that TMS over the cerebellum resets postural tremor but not resting tremor[1], we also performed a one-sided paired-samples t-test comparing these conditions. We primarily focused these analyses on the first tremor burst after TMS[1,10,11], but also report findings for the other four bursts to test whether tremor reset was transient or permanent (Table S-2). For postural tremor, we tested the *a priori* hypothesis that tremor reset is larger for the late condition than for the early condition, using a paired-sample t-test (one-sided). Fourth, we tested the effect of TMS over M1 or cerebellum on tremor power, separately for resting tremor and re-emergent tremor, using repeated measures ANOVA. For re-emergent tremor, we confined this analysis to the late condition, to circumvent interacting effects of wrist extension and TMS on tremor power (which prevented a stable baseline before the TMS pulse in the early condition). Thus, we performed a 2x2x9 interaction analysis with factors TREMOR TYPE (resting vs. postural tremor) x SITE (M1 vs. cerebellum) x TIME (baseline vs. eight post-TMS bins of 500 ms duration), with post-hoc t-tests (Tukey corrected).

SUPPLEMENTARY RESULTS

Tremor phenotype

All 14 patients had a re-emergent postural tremor, defined as a tremor with a similar frequency as resting tremor (delta frequency $\leq$1 Hz in all patients, no significant difference, Table S-1) and with a clear reduction in tremor power after a voluntary wrist extension. Across the group, wrist extension significantly reduced tremor power up to 3 seconds after movement onset (factor TIME, $F(1,20)=11.7$, $p<0.001$; part.$\eta^2=0.47$; post-hoc t-tests significant up to 3000 ms after wrist extension for five 500 ms intervals vs baseline, $p<0.05$ Tukey corrected).

Tremor reset index (Figure S-1)

TMS over M1 was more effective in resetting tremor than TMS over the cerebellum (SITE: $F(1,13)=155.4$, $p<0.001$; part.$\eta^2=0.92$), but this was not significantly different between resting tremor and re-emergent tremor (no SITE x TREMOR TYPE interaction, $F<1$). A post-hoc analysis showed that reset of both resting tremor and re-emergent tremor was not

modulated by bursts (F<1; Table S-2), indicating permanent resetting. A specific test of our *a priori* hypothesis confirmed that TMS over the cerebellum could reset re-emergent tremor but not resting tremor (paired-samples t-test, T(13)=2.1, p=0.026; Cohen's d=0.57; re-emergent tremor: TRI versus 0, T(13)=3.0, p=0.010; resting tremor: TRI versus 0, T(13)=1.0, p=0.33), confirming previous data[1]. Furthermore, for re-emergent tremor, the timing of the cerebellar TMS pulse (with respect to tremor onset) did not significantly influence the degree of tremor reset [T(13)=1.61, p=0.066, Cohen's d=0.43)]. A post-hoc analysis showed that reset of re-emergent tremor after cerebellar TMS decreased as a function of burst (factor TIME: F(4,52)=3.61, p=0.011; part.η^2=0.22; all values are shown in Table S-2), indicating transient resetting.

Tremor power (Figure S-2)

TMS over M1, but not the cerebellum, reduced tremor power in a similar way for resting tremor and re-emergent tremor (SITE x TIME interaction, F(8,104)=8.77, p<0.001, part.η^2=0.40; no 3-way interaction with TREMOR TYPE). More specifically, M1 stimulation significantly reduced resting tremor power up 1500 ms after the TMS pulse (TIME: F(8,104)=8.17, p<0.001, part.η^2=0.39; baseline vs. 0-500 ms, T(13)=4.9, p<0.001, baseline vs. 501-1000 ms, T(13)=6.0, p<0.001, baseline vs. 1001-1500 ms, T(13)=4.06, p=0.003, other bins not significant). A similar effect was seen for re-emergent tremor (TIME: F(8,104)=13.24, p<0.001, part.η^2=0.50, baseline vs. 0-500 ms, T(13)=5.7, p<0.001, baseline vs. 501-1000 ms, T(13)=5.2, p<0.001, baseline vs. 1001-1500 ms, T(13)=3.4, p=0.025, other bins not significant). In contrast, cerebellar TMS did not influence resting tremor power (TIME: F(8,104)=0.30, p=NS), or re-emergent tremor power (TIME: F(8,104)=1.33, p=NS).

SUPPLEMENTARY DISCUSSION

We investigated the role of M1 and cerebellum in controlling tremor rhythm and amplitude in resting tremor and re-emergent tremor in PD. There are two main findings. First, we confirmed a previous report that TMS over the cerebellum resets postural tremor, but not resting tremor[1]. We further qualify this previous finding by showing that this effect is present for electrophysiologically proven re-emergent tremor. This suggests that the cerebellum is part of the oscillator that controls the rhythm of re-emergent tremor. The

hypothesized difference between early and late stimulation (with respect to tremor onset) did not survive our statistical threshold (trend: p=0.066). Second, TMS over M1, but not the cerebellum, reduced tremor power of both resting tremor and re-emergent tremor for a period of up to 1.5 seconds. This suggests that M1 has a specific role in controlling the amplitude of Parkinson's tremor, regardless of the context in which it occurs (at rest or re-emergent postural tremor). Taken together, these findings suggest that tremor rhythm and amplitude are controlled by partly different nodes of the cerebello-(thalamo)-cortical circuit. Below we discuss these findings in more detail.

The role of the cerebello-cortical circuit in tremor rhythm

Our finding that TMS over M1 resets both PD resting tremor and postural tremor in a similar way is consistent with two previous studies[1,4], although only the more recent study specifically included patients with re-emergent tremor – as done here. There was permanent resetting, which suggests that the oscillator itself was affected by TMS[10,12]. These effects may be mediated by mechanisms within M1, or by brain regions that M1 projects to, such as the subthalamic nucleus or ventral intermediate nucleus (VIM) of the thalamus[13,14].

Our finding that cerebellar TMS resets re-emergent tremor, but not resting tremor, fits with a previous study comparing PD resting tremor with postural tremor[1]. It should be noted that the TRI reported here was smaller in magnitude as previously reported (0.1 vs. 0.5)[1]. Furthermore, the tremor reset we observed was transient, while it was found to be permanent in the previous study[1]. This difference may be related to the types of postural tremor included (re-emergent tremor in our study versus all postural tremors in previous work), to the stimulation intensity (56% of stimulator output in our study versus 68% in previous work), or a combination of these factors[1]. It is important to distinguish between different types of postural tremor in PD. We have previously shown that there are two different types of PD postural tremor: re-emergent tremor (81% of tremor-dominant patients) and pure postural tremor (19% of tremor-dominant patients)[6]. In an unselected PD sample that also includes non-tremor patients, 20-32% have re-emergent tremor [15]. These tremor types differed in terms of frequency, dopamine response, and timing with respect to the onset of the tremor, suggesting they have different pathophysiological substrates. Furthermore, although re-emergent tremor and resting tremor have been hypothesized to be a continuum, *i.e.* a "tremor of stability"[16], our findings add to converging evidence

showing that the tremor circuitry changes during posturing: the dopamine response of re-emergent tremor is smaller than that of resting tremor, and its frequency is slightly higher[6]. Our data suggest that these differences may be explained by the cerebellum, which comes in with voluntary movement, transiently modulates the oscillator of the tremor and possibly its frequency, while the fundamental character of the tremor remains unchanged.

The role of the thalamus was not investigated in this work, but the VIM is known to play a crucial role in PD tremor. Specifically, stereotactic surgery of the VIM is effective in reducing PD tremor[17,18], thalamic microstimulation in the VIM can reset the phase of PD postural tremor[19], and VIM-DBS at tremor frequency is able to entrain the ongoing PD tremor rhythm[20]. Our findings raise the question how these effects can be understood. Specifically, if the cerebellum plays only a transient modulatory role in re-emergent tremor and no causal role in resting tremor[1], then the effects of thalamic interventions cannot be explained (only) by an interruption of the cerebello-thalamo-cortical pathway. Anatomical studies in non-human primates suggest that, apart from the cerebellum, the VIM also receives dense projections from M1[13,14]. Furthermore, EEG studies in humans have shown that cortico-thalamic connectivity is altered in PD tremor compared to mimicked tremor[21]. This suggests that cortico-thalamic projections may play an additional role in PD tremor, explaining the differential contributions that VIM and cerebellum both have in PD tremor.

The TRI for cerebellar stimulation was not significantly different between the early (2 seconds after tremor onset) and late (10 seconds later) stimulation conditions. The trend-level effect in the hypothesized direction suggests that this null finding may be caused by a lack of power, or that more complex mechanisms are at play. Other studies have shown dynamic changes in the tremor circuitry during posturing in PD: the re-emergence of tremor has been associated with a time-locked excitatory event in M1[4]. Future studies may test the role of the cerebellum in tremor re-emergence.

The role of the cerebello-cortical circuit in tremor amplitude

Our finding that TMS over M1 reduced the power of resting tremor and re-emergent tremor in a similar way fits with previous data showing that an intervention (tACS over M1) reduced tremor amplitude in PD[22], while this effect was not observed for tACS over the cerebellum[15,33] or for VIM-DBS at tremor frequency[20]. This sets PD tremor apart from essential tremor, where a cerebellar lesion can reduce or even remove the tremor[23]. In our

data, TMS over M1 reduced tremor amplitude for 1.5 seconds. This is longer than the silent period [which has a duration of about 200 ms at the TMS intensity used here[10]], but shorter than the duration of tremor suppression after a voluntary movement (which was 3 seconds in our data). This suggests that brain regions involved in voluntary action planning may have an additional role in tremor suppression. For instance, the supplementary motor area has direct access to the pyramidal tract independent of M1[24,25]. Another possibility is that voluntary movements suppress tremor through the basal ganglia, which excite desired motor programs in M1 while inhibiting all others, including tremor [center-surround inhibition[26,27]]. Finally, post-movement synchronization in the beta range, which has been linked to reduced cortico-spinal excitability up to 1000 ms after voluntary movement, may play a role in the longer-lasting tremor suppression after voluntary movement[28,29].

The TMS pulse was given at an intensity that produces a MEP in the hand muscle. Therefore, the effect we observed may be driven in part by somatosensory afferences caused by a small hand movement. Indeed, median nerve stimulation can reset the rhythm of postural tremor in PD, but the duration of reduced tremor amplitude was much shorter than that observed here, *i.e.* in the range of the silent period (90-210 ms)[30]. Another study showed that mechanical perturbations (which change proprioceptive input) did not influence PD resting tremor[31], but it is unclear whether this difference is related to the perturbation or the tremor phenotype. Taken together, the close interactions between the motor and sensory systems, both at the central and at the peripheral level, make it difficult to attribute the effect we observed to one of the two systems.

Conclusion

We investigated the causal role of M1 and cerebellum in tremor rhythm and tremor amplitude, and we contrasted this contribution between PD resting tremor and re-emergent tremor. Our findings show that in PD, M1 is a key node that controls tremor rhythm and amplitude across tremor phenotypes. In contrast, the cerebellum has a very specific and probably transient role in controlling re-emergent tremor rhythm after movement onset, but it does not control the amplitude of either resting or re-emergent tremor.

SUPPLEMENTARY FIGURES

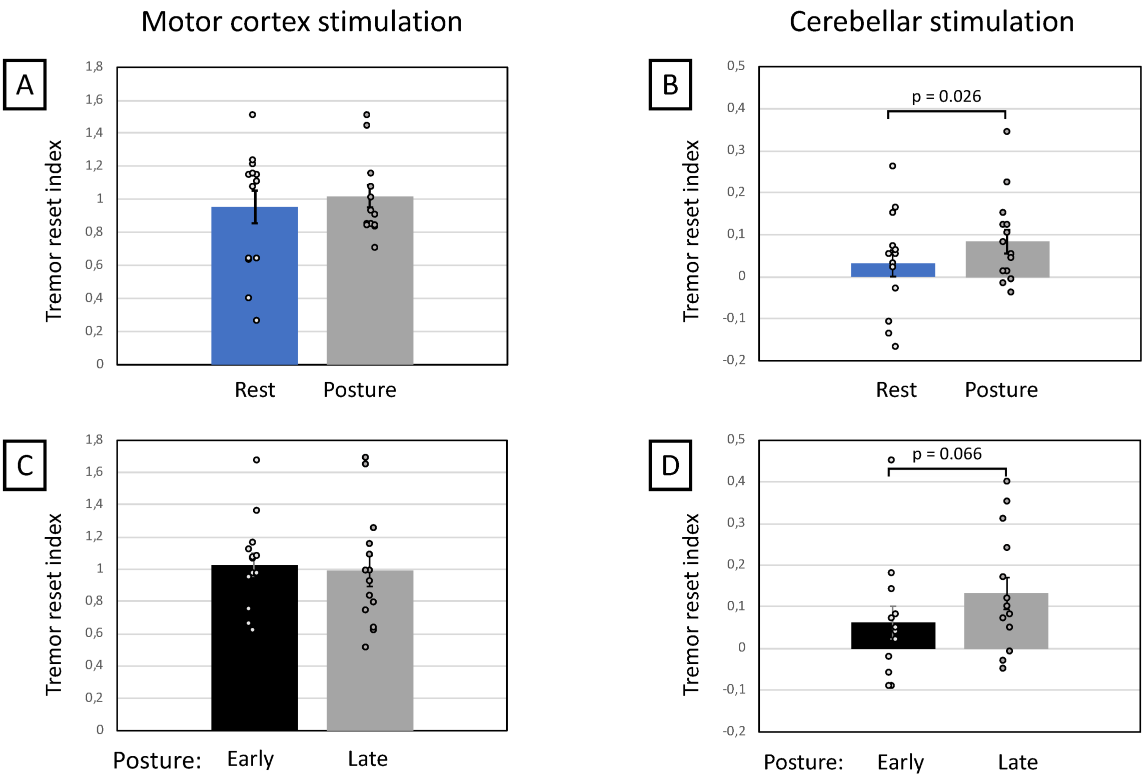

Figure S-1: Tremor reset indices

(A) Bar graph showing the mean and individual tremor reset indices (TRI) for M1 stimulation during rest and posturing. (B) Bar graph showing the mean and individual tremor reset indices for cerebellar stimulation during rest and posturing. (C) Bar graph showing mean and individual tremor reset indices for early and late stimulation over M1. (D) Bar graph showing the mean and individual tremor reset indices for early and late stimulation over the cerebellum. The asterisk (*) shows TRI's with a value of >2 standard deviations above the mean.

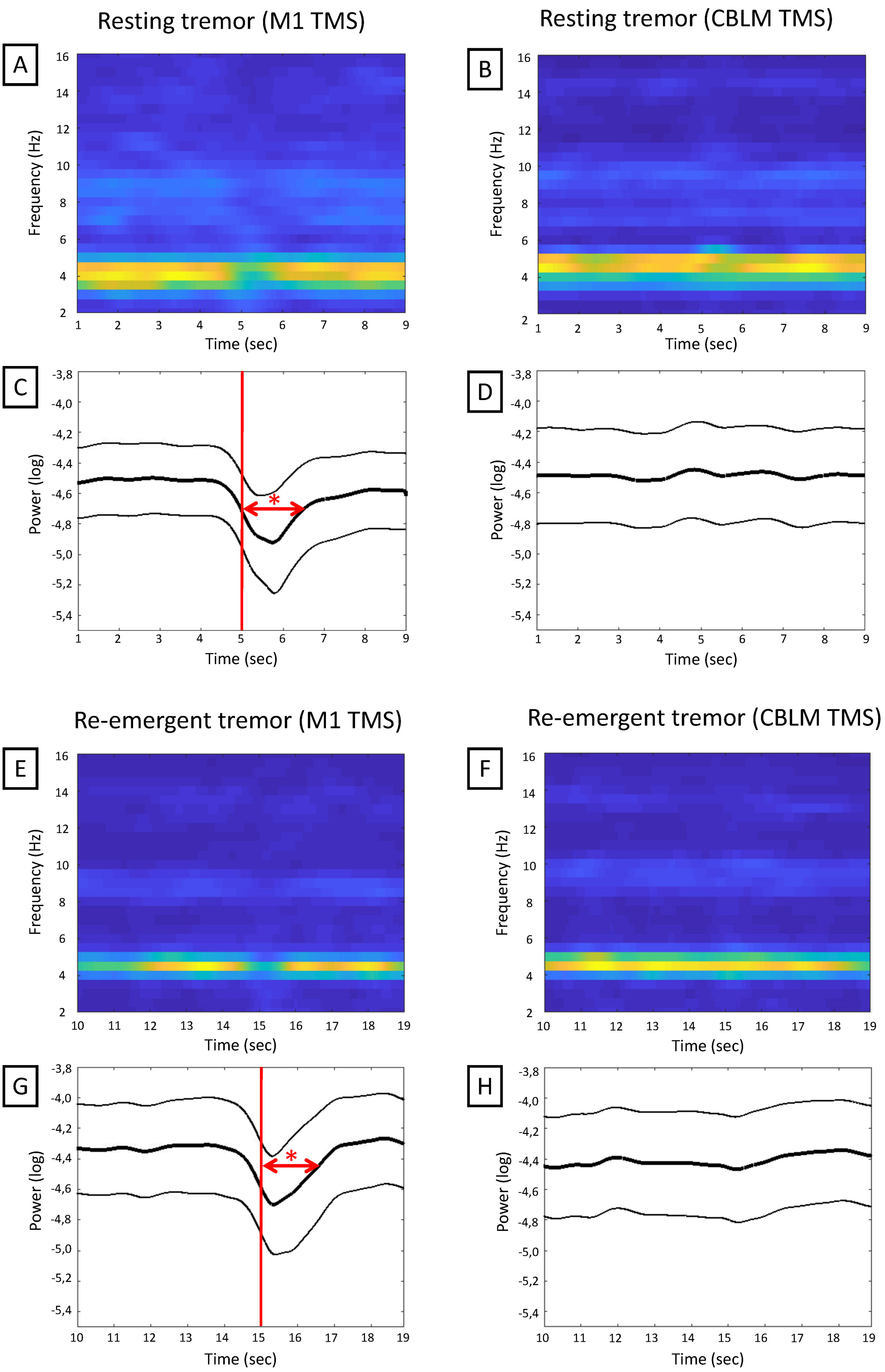
Resting tremor (M1 TMS)
Resting tremor (CBLM TMS)
A
B
C
D
Re-emergent tremor (M1 TMS)
Re-emergent tremor (CBLM TMS)
E
F
G
H
Frequency (Hz)
Power (log)
Time (sec)
*

Figure S-2: Effect of TMS on tremor power

Effect of TMS over the primary motor cortex (M1, left column) and cerebellum (CBLM, right column) on resting tremor (panels A-D) and re-emergent tremor (panels E-H). Panels A-B and E-F show the average time-frequency representation (TFR) of EMG tremor power (n=14). Panels C-D and G-H show the average (± SEM) tremor power (derived from EMG) over time at each patient's individual tremor frequency (n=14). Asterisks (*) indicate a significant drop in tremor power.

SUPPLEMENTARY TABLES

Table S-1: Clinical features

Patient	Age (yr)	Sex	Disease duration (yr)	UPDRS-III	Most affected side	Rest frequency (Hz)	Re-emergent frequency (Hz)	M1 stimulation (S50)	Cerebellum stimulation
1	65	M	3	29	R	4.4	4.4	53%	60%
2	59	F	14	38	L	4.6	4.4	60%	40%
3	67	M	7	25	R	4.2	4.4	42%	70%
4	49	M	3	54	R	5.0	4.8	47%	60%
5	70	F	10	29	R	4.0	5.0	59%	60%
6	63	F	0.5	25	R	4.6	4.8	-	-
7	61	F	20	30	L	4.8	5.0	47%	62%
8	58	M	5	56	R	5.2	5.4	56%	50%
9	74	F	10	13	R	6.2	5.2	56%	55%
10	58	M	4	64	R	4.4	4.4	70%	60%
11	75	M	3	54	L	4.4	4.0	42%	37%
12	59	F	4	19	R	5.2	5.6	80%	50%
13	51	M	9	33	R	4.6	5.2	65%	50%
14	35	M	8	33	L	4.6	4.6	75%	-
**Mean ± SD**	**60.3 ± 10.5**	**8M/ 6F**	**7.2 ± 5.2**	**35.9 ± 15.3**	**4L/10R**	**4.7 ± 0.5**	**4.8 ± 0.5**	**58% ± 12%**	**55% ± 9.5%**

Frequency values are calculated from the z-axis of the accelerometer. Some values could not be retrieved from patient records (indicated with “-“). Yr = year; M = male; F = female; R = right; L = left; S50 = stimulation intensity over M1; SD = standard deviation.

**Condition**	**Burst 1**	**Burst 2**	**Burst 3**	**Burst 4**	**Burst 5**
**Resting tremor**					
○ M1	**0.96** [0.76 - 1.15] (14)	**0.91** [1.20 - 0.63] (12)	**0.87** [1.20 - 0.54] (12)	**0.80** [1.17 - 0.43] (12)	**1.02** [1.31 - 0.72] (12)
○ Cerebellum	0.032 [-0.030 - 0.094] (14)	0.014 [-0.055 - 0.083] (14)	0.0057 [-0.056 - 0.067] (14)	-0.029 [-0.11 - 0.051] (14)	-0.086 [-0.22 - 0.047] (14)
**Re-emergent tremor**					
○ M1	**1.02** [1.15 - 0.89] (14)	**1.03** [1.24 - 0.82] (14)	**1.03** [1.28 - 0.77] (14)	**1.08** [1.43 - 0.74] (13)	**1.14** [1.56 - 0.71] (13)
○ Cerebellum	**0.084** [0.14- 0.029] (14)	0.0014 [-0.073 - 0.076] (14)	-0.051 [-0.19 - 0.086] (14)	-0.083 [-0.27 - 0.11] (14)	-0.18 [-0.41 - 0.057] (14)
M1 – early vs late					
○ Early	**1.03** [0.89 - 1.17] (14)	**1.03** [0.75 - 1.30] (13)	**1.02** [0.72 - 1.32] (12)	**1.07** [0.69 - 1.45] (12)	**1.21** [0.77 - 1.65] (11)
○ Late	**0.99** [0.80 - 1.18] (14)	**0.92** [0.65 - 1.19] (12)	**0.81** [0.54 - 1.08] (12)	**0.73** [0.44- 1.01] (12)	**0.58** [0.27 - 0.89] (12)
Cerebellum – early vs late					
○ Early	0.062 [-0.015 - 0.14] (13)	-0.039 [- 0.14 - 0.066] (12)	-0.08 [-0.26 - 0.099] (12)	-0.04 [-0.29 - 0.21] (12)	-0.14 [-0.44 - 0.16] (12)
○ Late	**0.13** [0.057 - 0.21] (14)	**0.11** [0.0082- 0.20] (13)	**0.22** [0.069 - 0.37] (12)	**0.21** [0.071 - 0.35] (12)	0.14 [-0.093 - 0.36] (12)

Table S-2: Tremor Reset Indices

Tremor reset indices were calculated for the first five bursts after the TMS pulse. The 95% confidence interval is shown within square brackets. Some participants did not have enough trials (at least 5 with clear EMG bursts) to calculate a reset index. The number of patients of which an index could be calculated with five or more trials is shown in round brackets. TRI’s in **bold** are significantly larger than 0.

REFERENCES

1. Ni Z, Pinto AD, Lang AE, Chen R. Involvement of the cerebellothalamocortical pathway in Parkinson disease. Ann Neurol 2010;68(6):816–824.

2. Muthuraman M, Raethjen J, Koirala N, et al. Cerebello-cortical network fingerprints differ between essential, Parkinson's and mimicked tremors. Brain 2018;73(Pt 1):69–12.

3. Volkmann J, Joliot M, Mogilner A, et al. Central motor loop oscillations in parkinsonian resting tremor revealed by magnetoencephalography. Neurology 1996;46(5):1359–1370.

4. Leodori G, Belvisi D, De Bartolo MI, et al. Re-emergent Tremor in Parkinson's Disease: The Role of the Motor Cortex. Mov Disord 2020;30:1591–11.

5. Oostenveld R, Fries P, Maris E, Schoffelen J-M. FieldTrip: Open source software for advanced analysis of MEG, EEG, and invasive electrophysiological data. Comput Intell Neurosci 2011;2011(1):156869–9.

6. Dirkx MF, Zach H, Bloem BR, et al. The nature of postural tremor in Parkinson disease. Neurology 2018;90(13):e1095–e1103.

7. Elble RJ. Tremor amplitude is logarithmically related to 4- and 5-point tremor rating scales. Brain 2006;129(10):2660–2666.

8. Panyakaew P, Cho HJ, Lee SW, et al. The Pathophysiology of Dystonic Tremors and Comparison With Essential Tremor. J Neurosci 2020;40(48):9317–9326.

9. Kukke SN, Paine RW, Chao C-C, et al. Efficient and reliable characterization of the corticospinal system using transcranial magnetic stimulation. J Clin Neurophysiol 2014;31(3):246–252.

10. Pascual-Leone A, Valls-Solé J, Toro C, et al. Resetting of essential tremor and postural tremor in Parkinson's disease with transcranial magnetic stimulation. Muscle Nerve 1994;17(7):800–807.

11. Pinto AD, Lang AE, Chen R. The cerebellothalamocortical pathway in essential tremor. Neurology 2003;60(12):1985–1987.

12. Britton TC, Thompson PD, Day BL, et al. Modulation of postural wrist tremors by magnetic stimulation of the motor cortex in patients with Parkinson's disease or essential tremor and in normal subjects mimicking tremor. Ann Neurol 1993;33(5):473–479.

13. Rouiller EM, Tanné J, Moret V, et al. Dual morphology and topography of the corticothalamic terminals originating from the primary, supplementary motor, and

dorsal premotor cortical areas in macaque monkeys. J Comp Neurol 1998;396(2):169–185.

14. Kultas-Ilinsky K, Sivan-Loukianova E, Ilinsky IA. Reevaluation of the primary motor cortex connections with the thalamus in primates. J Comp Neurol 2003;457(2):133–158.

15. Belvisi D, Conte A, Bologna M, et al. Re-emergent tremor in Parkinson's disease. Parkinsonism Relat Disord 2017;36:41–46.

16. Hallett M. Tremor: pathophysiology. Parkinsonism Relat Disord 2014;20 Suppl 1:S118–22.

17. Benabid AL, Pollak P, Gervason C, et al. Long-term suppression of tremor by chronic stimulation of the ventral intermediate thalamic nucleus. Lancet 1991;337(8738):403–406.

18. Schlesinger I, Eran A, Sinai A, et al. MRI Guided Focused Ultrasound Thalamotomy for Moderate-to-Severe Tremor in Parkinson’s Disease. Parkinson's Disease 2015;2015(1):1–4.

19. Milosevic L, Kalia SK, Hodaie M, et al. Physiological mechanisms of thalamic ventral intermediate nucleus stimulation for tremor suppression. Brain 2018;15(S3):1052–14.

20. Cagnan H, Little S, Foltynie T, et al. The nature of tremor circuits in parkinsonian and essential tremor. Brain 2014;137(Pt 12):3223–3234.

21. Muthuraman M, Heute U, Arning K, et al. Oscillating central motor networks in pathological tremors and voluntary movements. What makes the difference? NeuroImage 2012;60(2):1331–1339.

22. Brittain J-S, Probert-Smith P, Aziz TZ, Brown P. Tremor Suppression by Rhythmic Transcranial Current Stimulation. Curr Biol 2013;23(5):1–5.

23. Dupuis MJ-M, Evrard FL, Jacquerye PG, et al. Disappearance of essential tremor after stroke. Mov Disord 2010;25(16):2884–2887.

24. Chen S, Entakli J, Bonnard M, et al. Functional Corticospinal Projections from Human Supplementary Motor Area Revealed by Corticomuscular Coherence during Precise Grip Force Control. PLoS ONE 2013;8(3):e60291–11.

25. Entakli J, Bonnard M, Chen S, et al. TMS reveals a direct influence of spinal projections from human SMAp on precise force production. Eur J Neurosci 2013;39(1):132–140.

26. Mink JW. The basal ganglia: focused selection and inhibition of competing motor programs. Progress in Neurobiology 1996;50(4):381–425.

27. Beck S, Hallett M. Surround inhibition in the motor system. Exp Brain Res 2011;210(2):165–172.

28. Naros G, Grimm F, Weiss D, Gharabaghi A. Directional communication during movement execution interferes with tremor in Parkinson's disease. Mov Disord 2018;33(2):251–261.

29. Chen R, Yaseen Z, Cohen LG, Hallett M. Time course of corticospinal excitability in reaction time and self-paced movements. Ann Neurol 1998;44(3):317–325.

30. Britton TC, Thompson PD, Day BL, et al. Modulation of postural tremors at the wrist by supramaximal electrical median nerve shocks in essential tremor, Parkinson's disease and normal subjects mimicking tremor. J Neurol Neurosurg Psychiatr 1993;56(10):1085–1089.

31. Lee RG, Stein RB. Resetting of tremor by mechanical perturbations: a comparison of essential tremor and parkinsonian tremor. Ann Neurol 1981;10(6):523–531.
